# Urinary podocyte markers of disease activity, therapeutic efficacy, and long-term outcomes in acute and chronic kidney diseases

**DOI:** 10.1007/s10157-024-02465-y

**Published:** 2024-02-25

**Authors:** Akihiro Fukuda, Yuji Sato, Hirotaka Shibata, Shouichi Fujimoto, Roger C. Wiggins

**Affiliations:** 1 Department of Endocrinology, Metabolism, Rheumatology and Nephrology, Faculty of Medicine, Oita University, 1-1 Idaigaoka, Hasama-Machi, Yufu City, Oita 879-5593 Japan; 2 Division of Nephrology, Department of Internal Medicine, National Health Insurance Takachiho Town Hospital, Takachiho, Miyazaki, Japan; 3 Department of Medical Environment Innovation, Faculty of Medicine, University of Miyazaki, Miyazaki, Japan; 4 Division of Nephrology, Department of Internal Medicine, University of Michigan, Ann Arbor, MI USA

**Keywords:** Urinary podocyte, Podocyte injury, Glomerulosclerosis, mRNA biomarkers, Podocyte depletion hypothesis

## Abstract

A critical degree of podocyte depletion causes glomerulosclerosis, and persistent podocyte loss in glomerular diseases drives the progression to end-stage kidney disease. The extent of podocyte injury at a point in time can be histologically assessed by measuring podocyte number, size, and density (“Biopsy podometrics”). However, repeated invasive renal biopsies are associated with increased risk and cost. A noninvasive method for assessing podocyte injury and depletion is required. Albuminuria and proteinuria do not always correlate with disease activity. Podocytes are located on the urinary space side of the glomerular basement membrane, and as they undergo stress or detach, their products can be identified in urine. This raises the possibility that urinary podocyte products can serve as clinically useful markers for monitoring glomerular disease activity and progression (“Urinary podometrics”). We previously reported that urinary sediment podocyte mRNA reflects disease activity in both animal models and human glomerular diseases. This includes diabetes and hypertension which together account for 60% of new-onset dialysis induction patients. Improving approaches to preventing progression is an urgent priority for the renal community. Sufficient evidence now exists to indicate that monitoring urinary podocyte markers could serve as a useful adjunctive strategy for determining the level of current disease activity and response to therapy in progressive glomerular diseases.

## Introduction

Albuminuria and proteinuria are the mainstay biomarkers for the diagnosis and management of glomerular disease [1]. However, both glomerular and tubular injury, as well as physiologic stress, can cause increased protein excretion [2, 3]. Therefore, there is a need for additional markers that are specific for glomerular diseases, can accurately and reliably reflect both disease activity and response to treatment, and can predict time to end-stage kidney disease (ESKD).

## Podocyte depletion hypothesis

**Fig. 1:**
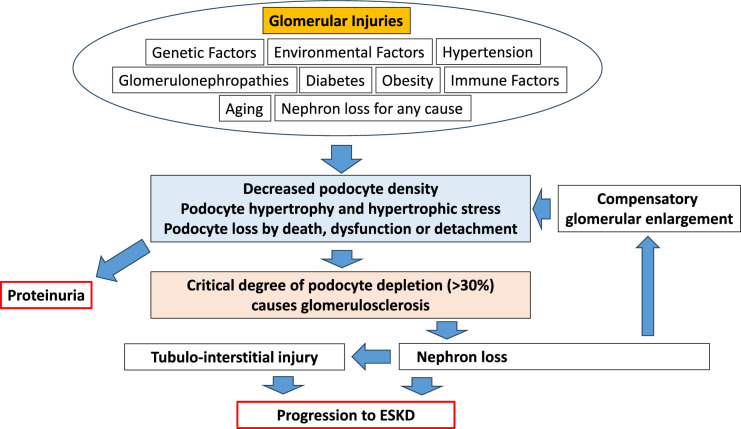
Podocyte depletion hypothesis. Podocyte injury and depletion have different causes in different glomerular diseases. The podocyte depletion hypothesis posits that loss of a critical proportion of podocytes (> 30%) by death, dysfunction or detachment causes proteinuria and glomerulosclerosis, and if persistent, progresses to ESKD

Podocytes are post-mitotic cells that possess limited or no capacity for replication after early post-uterine life. Therefore, in the event of loss of some podocytes, the remaining podocytes must adapt by hypertrophy to maintain complete foot process coverage of the glomerular basement membrane (GBM), thereby preserving normal filtration characteristics that minimize leakage of albumin and other proteins into the glomerular filtrate. The causes of podocyte injury and depletion vary among different glomerular diseases (Fig. 1). The podocyte depletion hypothesis proposes that the loss of a critical proportion of podocytes by death, dysfunction, or detachment results in proteinuria and glomerulosclerosis and can progress to ESKD if persistent.

### Genetic glomerular diseases caused by mutations in podocyte-expressed proteins

In 1999, the genetic cause of congenital nephrotic syndrome of the Finnish type was identified to be mutations in the NPHS1/nephrin gene, specifically expressed by podocytes and necessary for normal slit diaphragm function [4]. Since then, numerous other genetic variants expressed by podocytes have been shown to cause proteinuria and glomerulosclerosis in humans and in other experimental models [5]. These genetic data indicate that proteinuria and glomerulosclerosis are caused by the defective function of podocytes, which are key cells.

### Reduction in podocyte number per glomerulus

To confirm and extend this concept, transgenic rodent models have been used in our study as well as other studies to specifically deplete podocytes from glomeruli [6–9]. Proteinuria and glomerulosclerosis occur in a podocyte-specific manner and are directly proportional to the degree of podocyte depletion. Moreover, a phenomenon of glomerular destabilization was observed following a reduction in podocyte density (number per volume) by > 30%, which resulted in the glomeruli autonomously losing podocytes without further injury and passing through stages of focal segmental to global glomerulosclerosis with increasing podocyte depletion. Angiotensin II blockade was required to slow down or halt this autonomous process. Using more clinically relevant models of anti-GBM disease, we also demonstrated the quantitative relationship between podocyte depletion and the onset of proteinuria and progressive glomerulosclerosis [10]. These rodent podocyte depletion models demonstrate that proteinuria and further progression of glomerulosclerosis to ESKD are triggered by podocyte depletion itself.

### Glomerular volume enlargement can also cause podocyte hypertrophic stress and depletion, leading to ESKD

Increased glomerular volume (glomerular growth) can also result in reduced podocyte density, which may become critically reduced to a level below which podocytes are no longer capable of completely covering the filtration surface area with foot processes. A transgenic rat model in which podocytes specifically expressed a transgene (AA-4E-BP1) in the mTORC1 pathway that regulates growth was used for experimental confirmation of this concept. Podocyte-specific expression of this transgene resulted in reduced efficiency of mTORC1 growth signaling; thus, the transgenic podocytes failed to adapt effectively to cover the filtration surface area with foot processes under conditions of rapid glomerular enlargement. This resulted in some glomerular capillary loops becoming denuded of podocytes, thereby causing proteinuria and focal segmental glomerulosclerosis. Slowing down the glomerular growth rate (by reducing calorie intake, or the mTORC1 inhibitor rapamycin, or ACE inhibition) enabled podocytes to adapt successfully, thereby preserving the complete glomerular capillary loop coverage by podocyte foot processes and preventing the occurrence of proteinuria and glomerulosclerosis [11, 12]. In parallel, in more clinically relevant studies, the slowing down of glomerular growth rate by reducing calorie intake was demonstrated to similarly prevent critical podocyte depletion and development of proteinuria and glomerulosclerosis, using the Zucker diabetic rat model [13]. These models prove that accelerated glomerular growth itself can trigger the onset of proteinuria and progressive glomerulosclerosis, leading to ESKD.

## Summary

Podocytes are highly leveraged cells that completely cover the filtration surface area with foot processes that are necessary for maintaining the filtration barrier. Podocyte depletion caused by a reduction in podocyte number per glomerulus, an increase in glomerular volume, or defective podocyte function, can all trigger proteinuria and glomerulosclerosis. Persistent podocyte loss drives progressive glomerular diseases [6–21]. This “Podocyte Depletion Hypothesis” is now widely accepted as the fundamental mechanism underlying progressive glomerular diseases.

## Normal glomerular aging is associated with decreasing podocyte density.

The prevalence of ESKD is closely related to increasing age. The average glomerulus contains approximately 500 podocytes by shortly after birth [22, 23]. Approximately two podocytes are lost by the average normal glomerulus per year [24]. Glomeruli simultaneously rapidly enlarge after birth, maintaining normal kidney and body growth. Glomeruli continue to enlarge in part as a compensatory hypertrophic process resulting from nephron loss even after puberty, when normal body growth has ceased. Hence, with advancing age, the average podocyte density approaches a critical level (100 per um^3^) because of the combined effect of progressive reduction in podocyte number and increasing glomerular volume over time. Glomerulosclerosis supervenes when podocyte density exceeds the critical level. Younger glomeruli typically undergo focal and segmental sclerosis in response to podocyte depletion; however, older glomeruli undergo a rapid mass podocyte detachment process (“mitotic podocyte catastrophe”), resulting in global glomerulosclerosis [24]. Therefore, maintaining adequate podocyte numbers and preventing excessive glomerular enlargement are critical for the successful maintenance of renal function in older patients. Childhood glomerular diseases that cause podocyte loss early in life and common stressors of older age, such as obesity, diabetes, and hypertension, which cause glomerular enlargement and accelerated podocyte loss, increase the risk of critical podocyte depletion in older age, thereby accounting for the remarkably increased prevalence of ESKD in older age.

## Kidney biopsy podometrics

Kidney biopsy podometrics enable the estimation of podocyte number, density, glomerular volume, and other histological parameters, thereby providing a quantitative approach for identifying and monitoring podocyte depletion and treatment efficacy [25]. This methodology, together with machine learning and artificial intelligence, can enable the automated quantitation of kidney biopsy glomerular volume, podocyte number, size, and density, which can provide clinicians with the quantitative tools required to effectively prevent progression. [26].

## Urinary podometrics

The products of podocytes can be identified in urine when they detach or die because they are located on the urinary space side of the GBM. Several approaches, including enumerating urinary podocytes, podocyte-specific protein quantification, podocyte mRNA quantification, and quantitation of podocyte-specific extracellular vesicles (e.g., exosomes and migrasomes), have been used to measure podocyte products in urine (Table 1).

**Table 1:** Detection methods for urinary podocytes

Urinary source	Structural components	Detection methods
Sediment	Cells, casts and cell debris	Immunofluorescence, RT-PCR, ELISA assay, Western blot
Supernatant	Extracellular Microvesicles (exosomes, migrasomes)	

The urinary sediment obtained after centrifugation contains intact podocytes and other kidney cells derived from the lining of the urinary tract and cell debris (dead and dying cells). Stressed podocytes release subcellular elements that can also be detected by their podocyte-specific proteins.

### Urinary podocyte whole-cell enumeration

Podocalyxin, which is highly expressed on the apical cell surface of podocytes, and to a lesser extent by endothelial and other cells, is a sialic acid-rich negatively charged glycoprotein. Podocytes are the major podocalyxin-expressing cells that have direct access to the urinary tract; hence, podocalyxin-containing products in the urine are assumed to be podocyte derived. Using podocalyxin immunofluorescence, Hara et al. demonstrated that podocytes were shed in urine and an elevated number of podocalyxin-positive cells were observed in active and progressive glomerular diseases such as IgA nephropathy, post-infectious glomerulonephritis, and Henoch Schönlein Purpura (HSP) nephritis [27–29]. Using immunostaining for podocyte-specific proteins (podocin, nephrin, GLEPP1, and WT1), Shankland et al. demonstrated that podocyte cells were shed in urine in diabetic nephropathy and various glomerular disease animal models [30, 31]. Garovic et al. and other investigators demonstrated that podocytes identified by a podocin-specific antibody were identified in urine samples of women with pre-eclampsia and that the degree of podocyte loss was related to long-term renal function [32–35]. These reports show that podocyte cells in urinary sediment can be measured, and this can be a useful biomarker for assessing glomerular disease activity.

### Urinary sediment podocyte mRNA quantitation

Quantitative and specific evaluation of the amount of podocyte loss in the urine sample can be achieved by extracting RNA from urinary sediments and using RT-PCR to quantify mRNAs encoding podocyte-specific proteins (e.g., NPHS2/podocin) [7, 8]. This approach has high sensitivity and specificity, and multiple mRNAs can be simultaneously quantified.

#### Quality and stability of urinary sediment podocyte mRNA

The amount of RNA present in the urinary sediments decreased with time following urine sample collection and to plateau after approximately 12–24 h at approximately 60% of the initial value. However, if the urine sample was stored at 4 °C after voiding and processed within 24 h following collection, urinary sediment podocyte mRNA excretion was sufficiently well preserved for reliable quantitation and reproducibility with a coefficient of variation of approximately 35% [7, 36].

#### Potential utility of urinary sediment podocyte mRNA measurements

Excretion of urinary sediment NPHS2/podocin mRNA was demonstrated by rat podocyte depletion models to closely reflect glomerular disease activity [7, 8, 36]. Parallel human studies have also demonstrated increased urinary sediment podocin mRNA excretion in all glomerular diseases, which is directly related to disease activity [37–44].

#### Examples of application of the urine sediment podocyte mRNA method in the clinic

##### a. Alport syndrome (AS) (a genetic example)

AS is caused by mutations in the alpha3 chain of type IV collagen, a key component of the GBM. Defective podocyte adherence to the underlying GBM caused by these AS mutations results in accelerated podocyte detachment starting at birth when the alpha 3, 4, and 5 type IV collagen chains produced by podocytes replace the alpha 1 and 2 chains in the developing GBM. In classic cases of AS with major mutations, 21 years is the average age of reaching ESKD. Accelerated podocyte detachment (average 11-fold above control) was found to have begun early after birth and persisted long term. Podocyte detachment occurring at an 11-fold increased rate is predicted to cause critical podocyte depletion, resulting in glomerulosclerosis and ESKD by about 21 years of age. This genetic example illustrates the potential of urinary podocyte mRNA quantitation to predict the observed time to ESKD when the time of onset of the disease process is known [39].

##### b. Mild hypertension

Hypertension is a major cause of ESKD and is associated with progressive podocyte depletion and glomerulosclerosis leading to ESKD. Untreated mild hypertension (blood pressure in the high-normal range) is also associated with the development of ESKD in the elderly population [45]. Podocyte sediment mRNA was measured in extensively evaluated kidney donors, including a protocol biopsy at the time of transplantation, and who had entirely normal kidney function without proteinuria. However, subjects with blood pressure in the upper-normal range were observed to have a significantly higher rate of podocyte detachment (threefold) than those with blood pressure in the mid- and low-normal range. This accelerated podocyte detachment could be expected to cause critical podocyte depletion at advanced ages, which would account for the observed age-associated ESKD associated with mild hypertension. These data imply that a small increase in podocyte detachment rate, in this case due to mild hypertension, can have predictable long-term implications, which can manifest at advanced ages [41]. Furthermore, it is possible to noninvasively detect this long-term progression risk at an early age before detectable kidney injury is present.

##### c. Diabetic nephropathy

A prospective cohort study with 4 years of follow-up included 165 patients with type 2 diabetes (normal albuminuric [*n* = 94], microalbuminuric [*n* = 34], and proteinuric [*n* = 36]). Urinary sediment podocin mRNA was measured as a marker of podocyte loss. In the normal albuminuric group, urinary sediment podocyte marker excretion was significantly elevated compared with that in healthy age-matched subjects, thereby demonstrating its potential usefulness as a diagnostic marker earlier than microalbuminuria. In addition, in this normal albuminuric group, univariate and multivariate analyses of annual eGFR decline of ≥ 3 ml/min/1.73m^2^ as the renal outcome demonstrated that urinary sediment podocyte markers can predict future eGFR decline. In the normal albuminuric group, higher urinary sediment podocyte marker excretion levels at baseline were associated with the appearance of albuminuria four years later. Thus, the urinary sediment podocyte marker, but not albuminuria, was a predictor of eGFR decline in patients with eGFR ≥ 80 ml/min/1.73m^2^. This suggests that the urinary sediment podocyte marker may serve as a predictive marker of eGFR decline in type 2 diabetic nephropathy. These data are consistent with urinary sediment podocyte markers providing predictive information for type 2 diabetic nephropathy [43].

##### d. Acute glomerulonephritis that is self-limited and/or successfully treated

Urinary podocyte markers increase 100-fold in acute glomerulonephritis due to post-infectious glomerulonephritis or Henoch Schönlein Purpura (HSP), but return to baseline when the disease is in remission [37]. The urinary podocyte marker can thus detect disease activity and remission.

##### e. IgA nephropathy

Urinary sediment podocin mRNA excretion is significantly increased in IgA nephropathy compared to healthy control subjects. More severe injury associated with segmental sclerotic lesions (S lesions) and crescentic lesions (C lesions) is associated with higher urinary sediment podocin mRNA levels. However, patients who have been treated for more than one year with corticosteroids and renin–angiotensin system blockers show a significant reduction in urinary sediment podocin mRNA excretion. The urinary marker thus reflects disease activity and response to therapy [38, 44].

##### f. ANCA-associated glomerulonephritis and severe podocyte depletion in crescentic glomeruli

In patients with anti-neutrophil cytoplasmic antibody (ANCA)-associated nephritis, the urinary sediment podocyte mRNA marker level corresponded to the proportion of glomeruli with crescents, but only if the proportion of crescentic glomeruli was < 50%. In severe crescentic nephritis with > 50% crescents, urinary sediment podocin mRNA excretion, although still elevated, does not reflect the proportion of crescents, consistent with crescentic glomeruli having few podocytes remaining to detach and be detected in urine. These results indicate that under conditions of severe podocyte depletion, urinary podocyte markers do not reflect accumulated crescent formation and scarring [42].

## Summary

Based on these reports, urinary sediment podocyte mRNA excretion can be used as a prognostic biomarker in chronic diseases such as Alport syndrome, hypertension, and diabetic nephropathy, and to predict disease activity and response to therapy in acute/active glomerulonephritis.

### Detection of podocyte-derived subcellular structures in the urinary supernatant

In addition to detecting whole podocytes in urine, apical podocyte markers were used by Hara et al. (podocalyxin and GLEPP1) to identify microvilli-like structures shed from the apical cell surface of injured podocytes [46, 47]. These microvilli-like structures were measured in various human glomerular diseases, including IgA nephropathy, HSP nephritis, focal segmental glomerulosclerosis, and lupus nephritis, using an ELISA assay [46, 47]. Other previously conducted studies have demonstrated elevated podocalyxin protein levels in urine of patients with diabetic nephropathy without albuminuria or microalbuminuria [48], indicating that urinary supernatant podocalyxin protein levels may also serve as an early diagnostic tool for diabetic nephropathy. These reports indicate that urinary supernatant podocyte-associated proteins can serve as markers of podocyte stress and injury. No correlation was observed between urinary podocyte number and urinary supernatant podocalyxin in patients with IgA nephropathy, suggesting that different methods mark different biological processes [49].

### Exosomes in the urinary supernatant

Exosomes are 40–100 nm extracellular vesicles produced by all cells are mediators of near- and long-distance intercellular communication in health and disease. Pisitkun et al. isolated exosomes from human urinary supernatant, which contained the podocyte-associated proteins podocin and podocalyxin [50]. Exosomes containing WT1 and podocalyxin have been identified in urinary supernatants of various human glomerular diseases and animal models by western blotting and podocyte-specific mRNAs [51–53]. Abe et al. reported that podocyte-derived molecules from exosomes (e.g., WT1) serve as potentially useful markers for the early diagnosis and prognosis of diabetic nephropathy and other glomerular diseases [54]. Importantly, podocyte-specific markers are required for analysis because exosomes in urinary supernatants could have been derived from other kidney cells [55, 56]. Further research is required to clarify the potential clinical use of exosomes.

### Migrasomes in the urinary supernatant

Migrasomes (1um extracellular microvesicles), which are released by migrating cells and mediate intercellular communication, have been identified in the urinary supernatant [57, 58]. Cultured podocytes from humans and mice show elevated secretion of migrasomes following podocyte injury [59]. In a mouse model of PAN-induced renal injury, migrasomes in the urine supernatant were detected earlier than proteinuria [59]. Further research is required to define the potential utility of migrasomes in clinical applications.

### Podocyte marker detection in urinary sediment (cells) and supernatant (microvesicles) reflects different biology

**Fig. 2:**
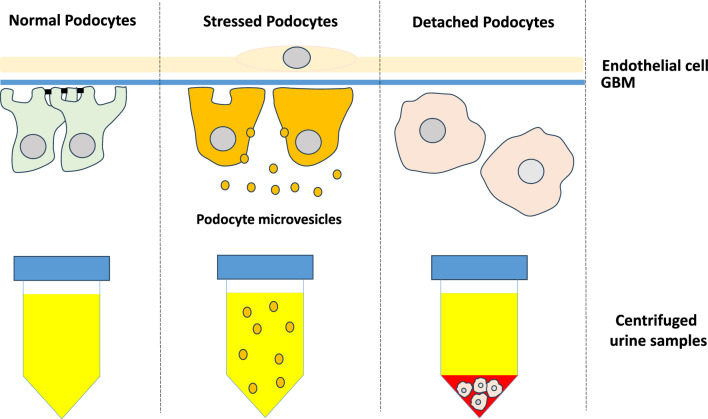
Mechanism of urinary podocyte excretion. Urinary sediment podocyte products (detached podocytes) reflect proliferative glomerular diseases associated with progression. In contrast, urinary supernatant podocyte products (stressed podocytes) reflect nonproliferative glomerular diseases, suggesting that different methods mark different biological processes

Podocyte markers in urinary sediment (podocin mRNA detected by RT-PCR method) and urinary supernatant (urinary supernatant podocalyxin protein level by ELISA) in patients with various kidney diseases were evaluated. Urinary sediment podocin mRNA (detecting cells) was significantly increased in proliferative glomerular diseases associated with more rapidly progressive loss of kidney function (IgA nephropathy with extracapillary proliferative lesions, ANCA-associated glomerulonephritis with crescent formation, and lupus nephritis type IV). In contrast, urinary supernatant podocalyxin (detecting microvesicles) was significantly elevated in nonproliferative glomerular injury with more stable renal function (membranous nephropathy and lupus nephritis with subepithelial dense deposition) [44] (illustrated in Fig. 2). The combination and appropriate application of podocyte markers in urinary sediment and supernatant could be useful adjuncts in the noninvasive diagnosis and management of glomerular disease activity; however, further study is required.

## Conclusion and future perspectives

Podocyte depletion due to podocyte loss, glomerular enlargement, hypertrophic stress, and accelerated detachment is the major mechanism underlying the progression of glomerular diseases (The Podocyte Depletion Hypothesis). A system of biomarkers that can measure this fundamentally important process is expected to be directly linked to outcome. This is in contrast to other downstream parameters, such as proteinuria, which may or may not be related to the outcome. Automated quantification of podocyte parameters in kidney biopsy (Biopsy Podometrics) is currently being developed for routine clinical application. Noninvasive measurement of podocyte-derived cells and/or subcellular particles in urine (Urinary Podometrics) at a particular point in time has now been demonstrated in group studies to be directly related to the degree of disease activity (glomerular injury) at that particular time [37, 38, 42], and especially in diabetes and hypertension, appears to be a more sensitive prognostic marker when compared with microalbuminuria [41, 43]. Furthermore, progression is associated with the persistent presence of podocyte products in urine, reflecting persistent podocyte injury and loss that results in accumulated podocyte depletion and progression corresponding to the rate of podocyte loss. Disease remission, either spontaneously or following treatment, is reflected by a reduction in urinary podocyte markers to baseline values. Urinary podocyte markers can thus potentially provide useful information to guide glomerular disease management, and thereby extend and complement the currently available biomarkers. Future challenges regarding clinical application include optimizing reproducibility and conducting prospective long-term trials to define predictive capacity for individualized decision-making using kidney biopsy podometric parameters as a quantitative and specific end-point.
